# Reevaluating the Value of (1,3)-β-D-Glucan for the Diagnosis of Intra-Abdominal Candidiasis in Critically Ill Patients: Current Evidence and Future Directions

**DOI:** 10.3390/jof11020091

**Published:** 2025-01-24

**Authors:** Emmanuel Novy, Mathieu Esposito, Anne Debourgogne, Claire Roger

**Affiliations:** 1 Department of Anesthesiology, Critical Care and Perioperative Medicine, Surgical Intensive Care Unit, CHRU-Nancy, F-54000 Nancy, France; mathespo@hotmail.com; 2 Université de Lorraine, SIMPA, F-54000 Nancy, France; anne.debourgogne@univ-lorraine.fr; 3 Mycology and Parasitology Laboratory, CHRU-Nancy, F-54000 Nancy, France; 4 UR-UM103 IMAGINE, Univ Montpellier, Division of Anesthesia and Critical Care, Pain and Emergency Medicine, Nîmes University Hospital, F-30029 Montpellier, France; claire.roger@chu-nimes.fr

**Keywords:** beta-D-glucan, diagnosis, biomarker, *Candida*, intra-abdominal candidiasis, critically ill

## Abstract

Intra-abdominal candidiasis (IAC) is associated with significant diagnostic and therapeutic challenges in critically ill patients. Traditional fungal cultures are slow, delaying appropriate antifungal treatment. (1,3)-β-D-glucan (BDG), a component of the fungal cell wall, has emerged as a potential biomarker for IAC, but its use in ICU settings is complicated by frequent false-positives results from invasive procedures and underlying conditions. This review examines the diagnostic value of BDG when present in serum and peritoneal fluid. While serum BDG is effective for excluding invasive fungal infections like candidemia, its specificity for IAC remains low in critically ill patients. Recent studies suggest that BDG levels in peritoneal fluid may provide better diagnostic accuracy, distinguishing IAC from bacterial peritonitis with higher specificity. We discuss the advantages, limitations, and practical aspects of BDG testing, emphasizing the potential of peritoneal BDG as a complementary tool. Further research is needed to refine diagnostic thresholds, validate its clinical utility, and establish the role of peritoneal BDG in improving timely, targeted antifungal treatment for IAC.

## 1. Definition and Conventional Diagnosis of Intra-Abdominal Candidiasis

### 1.1. Current Definition

Invasive candidiasis is the leading cause of fungal infections in intensive care units (ICUs) [1,2]. This category includes candidemia and deep-seated candidiasis, both of which are caused by hematogenous dissemination or direct inoculation of *Candida* into a normally sterile site [3]. Since the peritoneal cavity is considered to be sterile, the introduction of *Candida* following digestive tract disruption can lead to a diagnosis of deep-seated abdominal candidiasis.

Recently, the FUNDICU international consensus proposed a new definition for confirmed and possible intra-abdominal candidiasis (IAC) [4] (Table 1). This updated definition primarily affects the interpretation of peritoneal fluid culture. While the sampling conditions remain the same as those in the previous definition, a positive culture now indicates infection only if it is obtained outside the context of recent abdominal surgery or gastrointestinal perforation (<24 h). The authors argue that this change helps avoid misidentifying *Candida* colonization as infection. Additionally, unlike the previous definition, fungal antigen-based biomarkers such as (1,3)-β-D-glucan (BDG) are not included as criteria for diagnosing possible intra-abdominal candidiasis. This exclusion is due to two main reasons: the lack of standardized (1,3)-β-D-glucan use in clinical studies and the test’s low specificity, which is hindered by numerous false-positive results from confounding factors [4,5].

The prevalence of IAC ranges from 10% (in patients without specific risk factors for candidiasis) to 44% (in patients with complicated intra-abdominal infections or postoperative peritonitis) [2,6,7]. *Candida albicans* is the most frequently isolated species [8]. In 20–70% of cases, this infection is polymicrobial [7,9,10]. However, candidemia occurs in less than 5% of cases [2].

The overall mortality associated with IAC ranges from 20–60% [11]. Mortality is influenced by the quality of care (source control), the anatomical origin and extent of the infection (e.g., generalized peritonitis), and the patient’s comorbidities [12].

Given the high mortality rate and the diagnostic challenges in rapidly detecting *Candida*, antifungal treatment is often prescribed in ICU patients with severe intra-abdominal infections and risk factors for candidiasis [13,14]. This high level of antifungal use has contributed to increasing resistance to first-line antifungal agents, particularly echinocandins [15]. Consequently, research in this area is increasingly focused on developing diagnostic methods to accelerate *Candida* detection and/or promptly rule out its presence to avoid unnecessary antifungal exposure [5,16].

### 1.2. Microbiological Diagnosis of Intra-Abdominal Candidiasis

The gold-standard method for detecting *Candida* species relies on fungal culture. The main advantage of this approach is that it isolates the strain, allowing subsequent testing for antifungal susceptibility. However, its primary drawback is the culture time, which can range from 1 to 8 days depending on the inoculum size and the *Candida* species involved [17]. The time required to identify the yeast, once culture growth is detected, has been reduced to approximately 15 min with mass spectrometry. Therefore, the current challenge is early detection of *Candida* in peritoneal fluid to facilitate the timely initiation of empiric antifungal treatment.

Detecting *Candida* (or any microorganism) in peritoneal fluid is challenging due to several factors [18]:

- Low inoculum levels,
- Uneven distribution of viable cells within the sample,
- Variable fluid consistency, which can hinder certain analyses (particularly microscopic, colorimetric, or turbidimetric methods),
- Small sample volumes.

The current protocol in most centers begins with a direct examination of the peritoneal fluid. The fluid is then inoculated onto a yeast-supportive medium (such as Sabouraud or chromogenic agar specific to mycology). A retrospective study evaluating 152 cases of intra-abdominal infection reported a sensitivity of 35% and a negative predictive value of 78% for direct examination (for bacteria and yeast) of peritoneal fluid [19]. The authors noted that diagnostic performance was highly dependent on the experience of the examiner.

Owing to the limited sensitivity of direct examination and the time constraints associated with standard culture, the development of “nonculture-based” methods has attracted substantial interest. In addition to *Candida* PCR, which has been less well studied in the context of IAC, most research has focused on measuring (1,3)-β-D-glucan.

### 1.3. (1,3)-β-D-Glucan

(1,3)-β-D-Glucan (BDG) is a hydrophilic polysaccharide that maintains the fungal cell wall structure across various fungal species (including *Pneumocystis*), except for the *Mucorales* and *Cryptococcus* genera [20]. BDG is synthesized by bacteria, fungi, algae, and plants. However, it is not produced by mammals, where it is recognized by macrophages and dendritic cells, triggering the antifungal immune response [21].

BDG detection follows a process similar to that of bacterial lipopolysaccharide (LPS), utilizing the Limulus amebocyte lysate (LAL) cascade [22]. The LAL cascade can be activated through two independent pathways: bacterial endotoxin (using factor C) and BDG (via protein G). The final pathway is shared and involves a coagulation phase.

Currently available BDG assay kits use only the protein G pathway. Thus, a positive reaction indicates the presence of BDG in the sample. The coagulation time is detected using a photometric method, allowing for various threshold levels depending on the manufacturer (colorimetric or turbidimetric detection) [23].

The two most commonly used tests in daily routine practice are the “Fungitell^®^” and “Fungitell STAT^®^” assays produced by Associates of Cape Cod Inc. (Falmouth, MA, USA) and the “β-glucan test^®^” manufactured by Fujifilm Wako Chemicals Europe (Neuss, Germany). In clinical studies conducted in the ICU setting, the “Fungitell^®^” assay has been primarily evaluated. Both kits utilize the LAL cascade but differ in pretreatment and quantification methods, leading to specific detection thresholds. Pretreatment aims to inactivate potential inhibitors or activators (such as endotoxin) of the LAL cascade. To date, only blood testing has received FDA approval. Both manufacturers now offer single-sample tests to avoid the need to batch samples, which could delay turnaround times. For the U.S.-based manufacturer, the single-sample test is marketed as the “Fungitell Stat^®^” assay.

BDG is primarily eliminated by the liver [24]. An increase BDG half-life has been observed in cases of severe liver failure, particularly in pretransplant patients. BDG concentrations vary depending on the type of fungal infection and *Candida* species. *C. tropicalis* and *C. parapsilosis* produce the lowest BDG concentrations [25].

Thus, changes in serum BDG levels are influenced by multiple factors, including the fungal species, fungal burden, antifungal responses, host responses, and sample quality. These elements must be carefully considered to interpret test results reliably.

While serum BDG testing is now available in many microbiology laboratories, the measurement of BDG in peritoneal fluid remains limited for research purposes.

#### 1.3.1. Characteristics of BDG Testing

##### Serum BDG Testing

Serum BDG (sBDG) testing has been more extensively studied than peritoneal BDG (pBDG) testing since it has been validated for clinical use and serves as a broad detection tool for invasive candidiasis, especially candidemia. sBDG testing is integrated into clinical guidelines [26,27]. Indeed, when two measurements taken 48 h apart fall below the positivity threshold set by the manufacturer, guidelines suggest ruling out invasive candidiasis (albeit with a low evidence level). However, its positive predictive value is limited due to a high rate of false positives [18]. There are numerous sources of sBDG false positives in ICU patients [28]:

- infections: Certain bacteria, such as *Pseudomonas aeruginosa* and some streptococci, produce BDG. In other cases, such as enterococcal infections, elevated circulating BDG is secondary to increased intestinal transmembrane passage of BDG-producing species due to enterococcal sepsis.
- Certain therapeutic interventions: These include surgical sponges/drains, albumin supplementation, parenteral nutrition, and certain antibiotics (e.g., beta-lactams).
- Surgery: An increase in sBDG levels has been reported during and after digestive surgery (up to Day 5) without evidence of invasive candidiasis [29].
- Specific conditions or diseases: These include severe burns, chronic kidney disease, cystic fibrosis, and systemic lupus erythematosus.

Dialysis membranes, particularly older membranes, have long been considered a source of false-positive sBDG results. However, a recent retrospective study revealed that newer dialysis membranes are not associated with elevated sBDG levels [30].

The role of sBDG in optimizing the diagnosis of critically ill patients with suspected or confirmed IAC has yielded controversial results. One study reported no difference in the trajectory of postoperative sBDG levels between patients with and without IAC [31]. In contrast, Novy et al. reported lower sBDG levels in patients without IAC, regardless of the specific sBDG test used [7]. However, the limited availability of data prevents any definitive conclusions regarding its diagnostic utility in this context.

Studies specifically evaluating sBDG testing to optimize antifungal use by accelerating the diagnosis of invasive candidiasis have been conducted mainly in nonsurgical populations. Table 2 summarizes studies that assessed sBDG testing within an ICU-based antifungal prescription algorithm. Among these studies, (i) only one was multicenter [32], (ii) all included predominantly non-surgical, medical populations with very few cases of intra-abdominal candidiasis, and (iii) reported a low prevalence of invasive candidiasis, consisting mostly of candidemia. Thus, the use of sBDG to confirm invasive candidiasis or to guide antifungal initiation in ICU patients on the basis of sBDG levels is now clearly discouraged [5,33].

##### Peritoneal BDG Testing

Peritoneal BDG (pBDG) testing is based on the research hypothesis that sampling peritoneal fluid may yield fewer false positives. The first study to evaluate the utility of pBDG measurement was conducted in 2016 [37]. This retrospective, single-center study included 33 ICU patients. In the IAC group, the median pBDG level [Quartile 1–Quartile 3] was 1461 [325–5000] pg/mL, whereas it was 224 [68–1357] pg/mL in the control group (*p* = 0.03). The area under the ROC curve (AUC) for distinguishing IAC from nonfungal intra-abdominal infection was 0.76 (95% CI: 0.593–0.929, *p* = 0.036). The AUC value, with its associated confidence interval and *p* value, demonstrates the statistical robustness of the test’s discriminatory ability. The threshold used to calculate the AUC is equally important, as it defines the sensitivity and specificity that contribute to this performance measure. A negative predictive value (NPV) of 100% was achieved with a pBDG threshold of ≤310 pg/mL.

A second study was published in 2022 [31]: a prospective single-center trial involving 65 ICU patients with complicated intra-abdominal infections. In the IAC group, the median pBDG level was 2890 [942–12,326] pg/mL, whereas it was 1202 [317–4223] pg/mL in the control group (*p* = 0.135). The AUC for distinguishing IAC from other infections using pBDG levels was 0.62 (95% CI: 0.491–0.739, *p* = 0.11). The study also reported significant overlap in pBDG concentrations between groups for peritoneal samples.

The third study, published in March 2023 [38], was the first multicenter study to evaluate 113 ICU patients (135 peritoneal fluid samples). In the IAC group, the median pBDG was 8100 [3000–15,000] pg/mL, whereas it was 1901 [332–10,650] pg/mL in the control group (*p* = 0.002). The study required the dilution of 95 out of 135 samples because the pBDG values exceeded the upper limit of the assay. The AUC for pBDG in differentiating IAC from other intra-abdominal infections was 0.691 (95% CI: 0.606–0.767, *p* < 0.002), and an NPV of 100% was achieved with a threshold of 125 pg/mL. Interestingly, the authors also reported higher pBDG concentrations in peritoneal fluid with fecaloid characteristics. This finding could be attributed to the presence of polysaccharide components from feces that interfere with glucan detection. Additionally, fecaloid samples are often more pigmented, which may increase baseline light absorption during Fungitell^®^ kinetic spectrophotometric detection.

In summary, two studies yielded positive findings, whereas one was negative. Significant overlap in BDG concentrations between cases and controls was observed across both serum and peritoneal samples. These studies lacked sufficient power because of their small sample sizes and single-center designs, and the prevalence of intra-abdominal candidiasis was less than 30%. Additionally, all studies employed the “Fungitell Assay^®^”, Associates of Cape Cod (East Falmouth, MA, USA).

At the end of 2023, the most recent multicenter, prospective study on the evaluation of peritoneal 1,3 beta-D-glucan in critically ill patients with intra-abdominal candidiasis was published [7,39]. This study offers several advantages over previous studies, including a larger sample size of 199 patients, a higher incidence of intra-abdominal candidiasis (IAC) at 44%, and the use of the “β-glucan test^®^” manufactured by Fujifilm Wako Chemicals Europe (Neuss, Germany) for testing peritoneal fluid. The authors reported a median pBDG level of 447.7 [107.5–1578.0] pg/mL in the IAC group versus 133.1 [16.0–831.0] pg/mL in the control group (*p* = 0.03). Seventy-two samples exceeded the test’s upper quantification limit, and no dilutions were performed. When pBDG was used alone, the NPV was 82.3% at the 45 pg/mL threshold. When pBDG < 45 pg/mL was combined with sBDG < 3.3 pg/mL on Day 1, the NPV reached 100%. Thus, this prospective observational study demonstrated that combining low peritoneal BDG levels with low serum BDG levels improves the predictive value of the test. Specifically, very low pBDG levels strongly suggest the absence of *Candida* in the peritoneal cavity, and when paired with low serum BDG levels—despite potential serum false positives—this combination may more reliably exclude intra-abdominal candidiasis. Further validation is needed to confirm these findings.

Microbiologically, two additional insights emerged from this study:

- A comparison between mono-*Candida* and polymicrobial intra-abdominal infections (72% of patients) revealed no significant difference in pBDG levels, suggesting limited specificity due to bacterially induced false positives.
- Nine false negatives (pBDG < 45 pg/mL) were ultimately confirmed with positive fungal cultures. In these cases, the culture times exceeded four days, indicating a low inoculum and suggesting that pBDG levels correlate with fungal load in the peritoneal fluid.

Table 3 summarizes the main characteristics of studies that have assessed the utility of peritoneal BDG for diagnosing IAC in ICU patients. Importantly, all these studies were observational and noninterventional.

Thus, while a significant difference in pBDG values has been observed between patients with and without IAC, the current data highlight several limitations: (1) a high rate of false-positive results, as evidenced by the lack of significant difference between polymicrobial IAC and bacterial IAC; (2) technical challenges in measuring pBDG; and (3) the influence of fungal load. Finally, the impact of preoperative antifungal exposure on pBDG kinetics remains uncertain. In both the Nourry and Novy studies, no statistically significant difference in pBDG levels was detected between patients with or without antifungal exposure.

## 2. The Role of (1,3)-β-D-Glucan in the Management of Intra-Abdominal Candidiasis in Critically Ill Patients

First, it is important to note that only sBDG testing is included in guidelines, which recommend two tests taken 48 h apart to potentially discontinue empirical antifungal therapy [26,40]. On the basis of current data, if sBDG still holds value, it may have prognostic potential [41]. Indeed, the kinetics of sBDG may correlate with the course of infection in invasive candidiasis. A recent observational retrospective study demonstrated that a decrease in sBDG was associated with reduced mortality in critically ill patients with invasive candidiasis (34% of whom had IAC) [42].

For pBDG measurement, there are now sufficient observational data showing differences in pBDG concentrations between IAC and nonfungal intra-abdominal infections. However, the two largest multicenter prospective studies highlighted significant overlap in pBDG levels between single-species *Candida* infections and polymicrobial infections, as well as technical challenges in measuring pBDG in peritoneal fluid. Moreover, these were purely observational studies with peritoneal thresholds identified only for the specific populations studied, and these thresholds have not been validated in further research. For choosing between two BDG testing kits, no direct comparisons exist.

From a practical standpoint, access to sBDG testing varies across centers and countries. Two recent European surveys reported sBDG availability in more than 80% of centers [43,44]. The resulting turnaround times are also frequently over 24 h. Thus, before considering the routine use of pBDG testing alongside sBDG testing, several questions need to be addressed. Peritoneal thresholds that yield a negative predictive value (NPV) > 95%, depending on the test used, must be validated. Second, although the unit tests (β-glucan test^®^ and Fungitell STAT assay^®^) could theoretically reduce result turnaround times, integrating them into routine diagnostics requires reorganization to ensure faster daily results, for example, by implementing daily testing batches.

**Figure 1:**
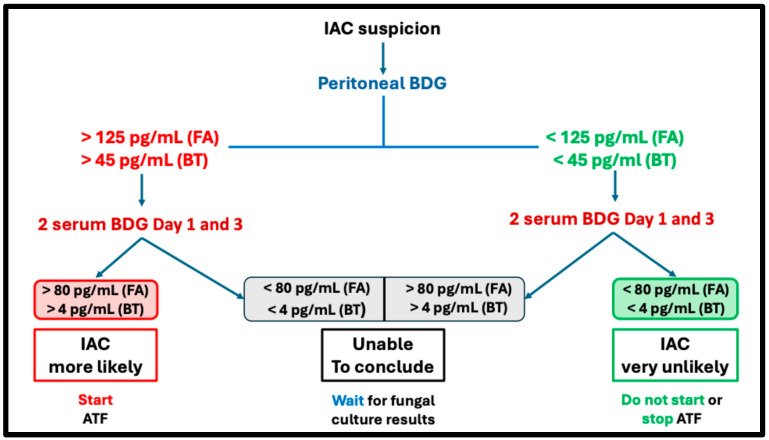
Proposed diagnostic algorithm for intra-abdominal candidiasis based on serum and peritoneal (1,3)-β-D-glucan: A literature-guided approach. Owing to the limited current literature, this algorithm should be interpreted in conjunction with clinical findings and other biomarker results. The serum and peritoneal BDG thresholds are derived from observational studies and have not yet been validated. Serum BDG must be measured postoperatively. Legend: ATF: antifungal; D: day; FA: Fungitell^®^ assay, Associates of Cape Cod, Inc. (Falmouth, MA, USA); IAC: intra-abdominal candidiasis; sBDG: serum (1,3)-β-D-glucan; BT: β-glucan test^®^, Fujifilm Wako Chemicals Europe (Neuss, Germany).

Table 4 summarizes current data on serum and peritoneal BDG testing and future considerations. Figure 1 proposes an algorithm integrating serum and peritoneal BDG testing when suspecting IAC.

## 3. Conclusions

The diagnosis of intra-abdominal candidiasis remains a complex challenge, requiring a balance between timely antifungal initiation and the minimization of unnecessary therapy. While serum BDG testing is widely available and FDA-approved, its diagnostic utility is limited in IAC because of high rates of false positives and moderate NPVs. Peritoneal BDG testing offers a promising alternative, with emerging evidence suggesting its utility in excluding IAC in high-risk patients. However, significant hurdles, including a lack of external validation, overlapping BDG levels between fungal and bacterial infections, and technical inconsistencies, limit its current clinical applicability.

To increase the utility of (1,3)-β-D-glucan in the management of IAC, further large-scale, multicenter studies are needed to establish robust diagnostic thresholds and validate the clinical performance of different BDG assays. However, the practical implementation of BDG testing, especially in peritoneal fluid, presents challenges. The availability of BDG assays, turnaround times for results and the complexity of performing assays on peritoneal fluid—where values may be difficult to measure—pose significant barriers to integrating these tests into routine clinical practice. Consequently, until these logistical and technical hurdles are addressed, the use of BDG, particularly in peritoneal fluid, should be limited to research settings or as an adjunct in a more comprehensive diagnostic workflow.

## Figures and Tables

**Table 1:** Current definition of intra-abdominal candidiasis according to the FUNDICU consensus [4].

Confirmed Intra-Abdominal Candidiasis	Possible Intra-Abdominal Candidiasis
1. Histological evidence of *Candida* in a sample or biopsy from a normally sterile site, confirmed by culture or PCR	1. Presence of >1 clinical or radiological criteria of intra-abdominal infection
2. Positive culture from a peritoneal fluid sample (direct puncture, surgical sample, or sample from an abdominal drain placed within <24 h) AND clinical or radiological signs of intra-abdominal infection in a patient without recent gastrointestinal perforation or abdominal surgery	2. Positive culture from a peritoneal fluid sample (direct puncture, surgical sample, or sample from an abdominal drain placed within <24 h) in a patient where source control occurs >24 h after gastrointestinal perforation, anastomotic leak, or tertiary peritonitis
3. Positive blood culture for *Candida*	
*Any one of these criteria is sufficient for diagnosis*	*Both criteria are required*

**Table 2:** ICU studies using serum BDG in an algorithm to guide the initiation or discontinuation of empirical antifungal treatment.

Author, Year	Study Type and Population	BDG Test	Algorithm	Invasive Candidiasis Prevalence	Antifungal Exposure
Rouzé, 2017 [34]	Single-center, Prospective RCT N = 109 (27% surgical patients)	Fungitell^®^	Discontinuation of antifungal treatment guided by sBDG vs. clinician’s judgment	5% (N = 6) Prevalence of IAC not reported	Fewer days on antifungals (BDG group)
De Pascale, 2020 [35]	Single-center, Prospective RCT N = 108 (26% surgical patients)	Fungitell^®^	Discontinuation of antifungal treatment guided by sBDG vs. clinician’s judgment	10% (N = 11) Prevalence of IAC 2% (N = 2)	Fewer days on antifungals (BDG group)
Erb, 2023 [36]	Single-center, Prospective RCT N = 41, mixed medical-surgical (cardiovascular focus) 50/50%	Fungitell^®^	Discontinuation of antifungal treatment guided by sBDG + mannans vs. clinician’s judgment	22% (N = 9) Prevalence of IAC not reported	No difference in antifungal days between groups
Bloos, 2022 [32]	Multicenter, Prospective RCT N = 339 (Sepsis)	β-glucan test^®^	Antifungal initiation guided by sBDG vs. culture	14% (N = 48) Prevalence of IAC not reported	Increased antifungal use (BDG group)

Legend: BDG: (1,3)-β-D-glucan; IAC: intra-abdominal candidiasis; RCT: randomized controlled trial; sBDG: serum (1,3)-β-D-glucan.

**Table 3:** Peritoneal (1,3)-β-D-glucan evaluation in suspected and confirmed intra-abdominal candidiasis.

Author, Year	Study Type	Population	IAC Prevalence	BDG Test	pBDG IAC+	pBDG IAC-	pBDG Threshold **
Novy, 2018 [37]	Single-center Retrospective	N = 33 Nosocomial Peritonitis	20% (n = 7)	Fungitell^®^	1461 *	224	310
Dupont, 2022 [31]	Single-center Prospective	N = 65 Nosocomial Peritonitis	30% (n = 19)	Fungitell^®^	2890	1202	NE
Nourry, 2023 [38]	Multicenter Prospective	N = 113 Peritonitis	25% (n = 28)	Fungitell^®^	8100 *	196	125
Novy, 2023 [7]	Multicenter Prospective	N = 199 Peritonitis	44% (n = 87)	β-glucan test^®^	447.7 *	133.1	45

Legend: IAC: intra-abdominal candidiasis; NE: not evaluated; pBDG: peritoneal (1,3)-β-D-glucan (pg/mL); * statistically significant difference; ** threshold below which intra-abdominal candidiasis can be ruled out.

**Table 4:** Current standings and future considerations of serum and peritoneal (1,3)-β-D-glucan.

Serum (1,3)-β-D-Glucan	Peritoneal (1,3)-β-D-Glucan
**Current standings**	
FDA/EMA-approved assay Affordable cost Increasing availability of assays	Only observational data available No FDA/EMA approval
**Key considerations and limitations**	
High rate of false positives NPV 65–80% Turnaround time > 24 h Prognostic value in absence of downslope of sBDG overtime	Research-use-only assay Many results exceed the upper limit of quantification, complicating interpretation High rate of false positives NPV 82–100% Thresholds identified but lack external validation
**Proposed recommendations**	
Serum and peritoneal BDG should not be used in isolation but rather integrated into a diagnostic algorithm that incorporates clinical scoring systems and other biomarkers	
Consideration of assay manufacturer to interpret BDG results (Fungitell^®^ vs. β-glucan test^®^) is essential	
Only the negative predictive value should be considered	
**Key areas for investigation and improvement**	
Challenges persist in interpreting peritoneal supra-threshold values	
No comparative studies are available between both manufacturers	
No data exist for peritoneal samples using the Associate of Cape Cod unitary test (Fungitell Stat^®^)	
Influence of antifungal exposure on peritoneal BDG remains uncertain	
**Research agenda**	
To validate the diagnostic performance of peritoneal BDG, alone or in combination with serum BDG in additional cohorts of critically ill patients with intra-abdominal infections	

## Data Availability

Data sharing is not applicable.
